# Effects of Linpan nature therapy on health benefits in older women with and without hypertension

**DOI:** 10.3389/fpubh.2023.1208481

**Published:** 2023-11-02

**Authors:** Xiang Ye, Zhiwen Dou, Mingyan Jiang, Zhenghua Luo, Mao Li, Haixiong Tang, Xiao Huang, Yuqian Wang, Liwei Dong, Xiaoguang Mao, Yu Feng

**Affiliations:** ^1^College of Landscape Architecture, Sichuan Agricultural University, Chengdu, China; ^2^Railway Cultural Tourism Investment Group, Health Industry Co., Ltd., Chengdu, China

**Keywords:** nature therapy, older women, hypertension, physiological response, sleep quality

## Abstract

**Background:**

Nature therapy can significantly benefit the physiology and psychology of middle-aged and older people, but previous studies have focused on forest environments. The restoration potential of rural environments in urban fringe areas, which are more accessible to older people on a daily basis, has not been fully studied. This study assessed the effects of nature therapy on the physical and mental health of older women in a rural setting (locally known as Linpan) in the urban fringe area of Chengdu, China.

**Methods:**

We recruited a total of 60 older women (65.3 ± 5.5 years old) living in cities for 3 days of nature therapy in the winter (30 subjects) and spring (30 subjects), including 20 hypertensive patients.

**Results:**

The results showed that the overall blood pressure, pulse and sleep dysfunction rating scores of the participants were significantly lower than the pretest levels, and the finger blood oxygen saturation, mid-day salivary alpha-amylase and cortisol were increased post-treatment. Increases in these biomarker indicates and increase in stress. There were significant differences in the changes in systolic blood pressure between the hypertension group (HTN) and the normal group (normal) (HTN decreased by 8.8%, normal decreased by 5.4%), salivary alpha-amylase content (HTN decreased by 0.3%, normal increased by 16.9%), and sleep dysfunction rating scores (HTN decreased by 59.6%, normal decreased by 54%). The decreases in systolic blood pressure and pulse in the winter group were higher than those in the spring group by 1.8 and 4.4%, respectively, while the increases in salivary alpha-amylase content and salivary cortisol content were lower than those in the spring group by 11.7 and 11.2%, respectively, and the decrease in sleep dysfunction rating scores was lower than that in the spring group by 7.1%.

**Conclusion:**

Our study concluded that nature therapy based on various health activities in the Linpan has significant health effects on older women. It can regulate blood pressure and pulse in older women, relieve cardiovascular disease, improve sleep quality. Meanwhile, older women with high blood pressure experienced a more significant effect than the healthy group.

## 1. Introduction

With the spread of subhealth and a declining birth rate, China has the largest population older people in the world (1). According to the seventh national census released in 2021, 18.7% of China’s total population is above the age of 60 (2). Research results showed that the prevalence of hypertension in Chinese residents aged over 60 years was 58.3% (3), the total prevalence of sleep disorders was 35.9% (4), and the total number of patients with dementia and mild cognitive impairment (MCI) was more than one-fifth of the total number of people over 60 years old (5). The incidence of chronic diseases and multiple diseases among the older people in China is higher, and the incidence of chronic diseases among older women is higher (6).

### 1.1. Study of modern nature therapy

Studies have found that exposure to nature can improve health in older people. Short exposure to tree-covered urban parks in the older people has a protective effect on cardiovascular health (7). A virtual natural environment can also promote the psychological recovery of middle-aged and older people and motivate them to experience nature outdoors (8). Heart rate and blood pressure in middle-aged and older people were improved after a 2 hour forest immersion, and they became more relaxed (9). Another study with middle-aged women found that participants’ physical and mental health also improved after forest therapy (10). Forest therapy is a promising treatment strategy for older people with chronic diseases. It can lower blood pressure to the optimal range and prevent hypertension, thereby reducing the risk of cardiovascular and kidney disease in the older people (11, 12). The time of exposure to nature in the above studies varies from tens of minutes to several hours, and the result of such short-term exposure to nature may be an acute reaction. Some studies suggest that the blood pressure lowering effect of forest therapy will not last long, and long-term frequent exposure may have a better impact on physical and mental health (13).

The pattern of living in forests for days or more resulted in more health benefits for middle-aged and older people. Studies have shown that 3 days of forest therapy improves physical and mental health and creative performance in middle-aged and older people, which can improve their cognitive function (14) and reduce cognitive decline and psychological and physical risk factors and may be applicable to the prevention of Alzheimer’s disease (15). For people with chronic diseases, forest therapy for 3 days or more can assist in the treatment of cardiovascular diseases such as hypertension and chronic heart failure in the older people (16, 17). Longer periods of nature therapy allows for monitoring of sleep improvement. The study found that 6 days of forest therapy may help improve sleep quality in patients with gastrointestinal cancer (18). Sleep disorders in the older people usually originate from other chronic diseases (19), and improving sleep disorders may have therapeutic effects on chronic diseases.

### 1.2. Linpan

**Figure 1:**
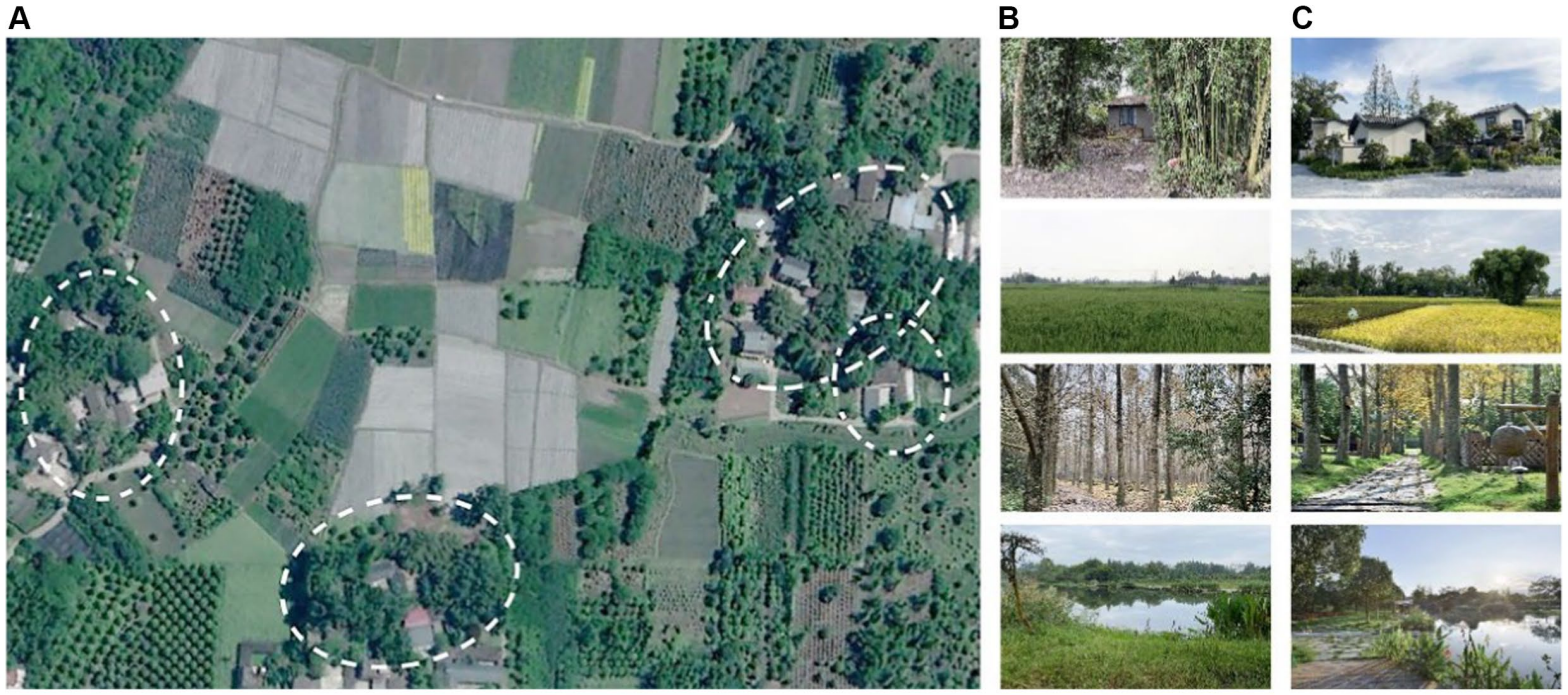
Linpan view in Chengdu Plain. **(A)** Linpan settlement and units (circled) (base map from Google Earth); **(B)** examples of original views; **(C)** examples of ecological scenic spots for cultural tourism.

The research areas in the field of nature therapy mostly focus on forests, but forests are inconvenient for urban residents, especially older people, to access on a daily basis. The Linpan settlement in western Sichuan (Linpan for short) is a large area of villages around the cities in the Chengdu Plain, where the basic unit consists of peasant houses surrounded by a large number of trees, canals and fields, and the tree canopy coverage of Linpan ranges from 60–85%. Most Linpan units are usually 50–100 m apart and vary in size from 0.2–7 hm^2^, which is an important part of the local agroforestry ecosystem (20, 21). In China, Linpan has been prioritarily constructed as ecological scenic spots for cultural tourism because of its unique spatial environment for human habitation, it has gradually become a destination for people to escape from the urban environment (Figure 1). We believe that Linpan, which is located on the fringes of the city, can also be used as a natural environment for nature therapy. Although it does not have the physical and chemical benefits of a large amount of forest vegetation, it is more accessible on a daily basis, has more options for activities, and the diversity of landscape space is higher than that of forest space.

### 1.3. Nondrug therapy in other natural environments

In addition to forest therapy, nondrug therapy in other natural environments is also beneficial to the physical and mental health of middle-aged and older people. Studies have found that different gardening activities improve the physical and mental health of older people, and the activities of direct contact with plants will have a more significant impact on the health of the older people (22). Long-term adherence to Baduanjin and Tai Chi exercises can relieve musculoskeletal pain and improve sleep quality in patients with chronic diseases and is regarded as a behavioral therapy for insomnia (23, 24). Additional weekly physical activity in older people with memory impairment can modestly improve cognitive performance (25). In addition, music therapy may also improve the emotional health of people with mild Alzheimer’s disease (AD) (26). Therefore, for the Linpan nature therapy selected in this study, in addition to direct exposure to nature, we implemented different overlapping activities to provide the older people with a more accessible nature therapy site.

## 2. Materials and methods

### 2.1. Participants and study area

We recruited 60 female participants (mean age: 65.3 ± 5.5 years) with the following characteristics by distributing leaflets and basic health information questionnaires: (1) over age 55 (8 aged 55–59, 40 aged 60–69, and 12 aged 70–79, Table 1), (2) no cognitive and communication difficulties, (3) normal vision and hearing condition, and (4) city living, not living alone. Table 1 reports the experimental grouping of participants. Female participants were selected to rule out the effects of men being more likely to smoke and drink during the experiment, and none of them had smoking, drinking, or coffee habits. This study was approved by the local Ethics Committee of the College of Landscape Architecture, Sichuan Agricultural University, China (protocol code SICAU201504120023 and date of 8 April 2015 approval).

**Table 1:** Overview of sample grouping characteristics and pre-intervention blood pressure (systolic, SBP; diastolic, DBP) and sleep dysfunction rating scores (SDRS) (*N* = 60).

Variable	Group	*N*		Age (range) (years)		SBP (mmHg)		DBP (mmHg)		SDRS (score)	
				Mean ± SD	*P*	Mean ± SD	*P*	Mean ± SD	*P*	Mean ± SD	*P*
Education	Primary	5		69.8 ± 4.0	0.016*	131.6 ± 21.7	0.832	72.4 ± 11.5	0.666	10.6 ± 3.8	0.167
	Middle	19		66.9 ± 5.0		129.7 ± 11.6		77.5 ± 7.9		11.4 ± 4.8	
	High	32		63.4 ± 5.6		126.8 ± 14.9		76.1 ± 8.4		8.4 ± 5.0	
	College	4		67.8 ± 1.0		125.8 ± 12.5		77.8 ± 5.9		12.0 ± 6.2	
Gender	Female	60	8	57.0 ± 1.6 (55–59)	0.000**	128.1 ± 14.2		76.4 ± 8.3		9.8 ± 5.0	
			40	64.5 ± 2.7 (60–69)							
			12	73.6 ± 2.8 (70–79)							
Season	Winter	30		65.6 ± 5.3	0.745	125.5 ± 15.3	0.168	75.0 ± 9.6	0.211	9.9 ± 5.5	0.899
	Spring	30		65.1 ± 5.7		130.6 ± 12.6		77.7 ± 6.6		9.7 ± 4.6	
Health condition (Long-term medication)	Normal (No)	40		65.0 ± 4.8	0.502	124.1 ± 12.5	0.002**	74.9 ± 8.6	0.047*	8.8 ± 4.8	0.031*
	Hypertension (HTN) (Yes)	20		66.1 ± 6.8		136.0 ± 14.3		79.4 ± 7.0		11.8 ± 5.0	
Season × Health condition	W-Normal	20		65.6 ± 4.6	0.719	122.3 ± 14.6	0.006**	73.5 ± 10.6	0.136	8.2 ± 4.4	0.064
	W-HTN	10		65.5 ± 6.8		132.1 ± 15.5		78.1 ± 6.8		13.3 ± 6.1	
	S-Normal	20		64.3 ± 5.0		126.0 ± 10.0		76.3 ± 5.9		9.5 ± 5.2	
	S-HTN	10		66.7 ± 7.1		139.9 ± 12.6		80.6 ± 7.3		10.2 ± 3.2	

Health condition only includes hypertension, all participants have no other underlying diseases. *p < 0.05 and **p < 0.01.

**Figure 2:**
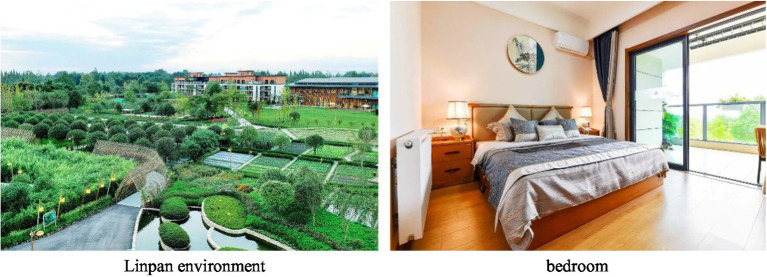
Linpan environment and bedroom.

The Linpan study area is located in Pidu District, Chengdu, China (30°49′28″ north, 103°49′38″ east, about 35 km from downtown Chengdu), which is a suburban nursing center for older people. The total area of this Linpan community is approximately 73.33 hm^2^, including approximately 5.78 hm^2^ for the older people care community, 1.24 hm^2^ for medical institutions and 2.68 hm^2^ for the older people care industry base. Participants had daily activities within the total area and lived in the community for two nights (Figure 2).

### 2.2. Study design

**Figure 3:**
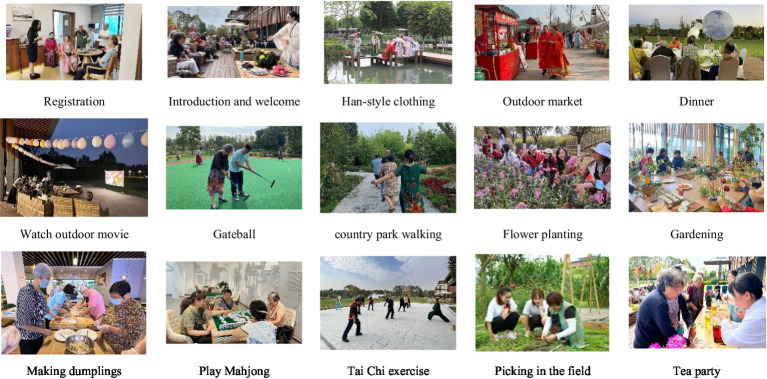
Activities.

We conducted a study in the winter of 2021 and spring of 2022, each lasting 2 days and two nights, from the first day after lunch to the third day after lunch. The relevant schedule and photos of activities are shown in Table 2 and Figure 3. On the first morning of the study, participants got up and waited at home (45–60 min drive from the study site) for a unified vehicle to take them to the study site. Arrival was followed by a check-in and rest period, during which the participant’s travel stress was relieved (in a unified environment).

**Table 2:** Time schedule of various activities during Linpan nature therapy.

Day	Time	Program
Day 1	9:00–10:30	Registration
	10:30–11:30	Pretest
	11:30–14:30	Lunch break
	14:30–16:00	Introduction and welcome
	16:00–17:00	Han-style clothing
	17:00–18:00	Outdoor market
	18:00–19:30	Dinner
	19:30–21:00	Watch outdoor movie
	21:00–22:00	Free time and sleeping
Day 2	8:00–9:00	Breakfast
	9:00–9:30	Gateball
	9:30–11:30	Country park walking
	11:30–14:30	Lunch break
	14:30–15:30	Flower planting
	15:30–17:00	Gardening
	17:00–18:00	Making dumplings
	18:00–19:30	Dinner
	19:30–21:00	Play Mahjong
	21:00–22:00	Free time and sleeping
Day 3	8:00–9:00	Breakfast
	9:00–9:20	Tai Chi exercise
	9:20–9:50	Picking in the field
	9:50–10:20	Tea party
	10:20–10:30	Break
	10:30–11:30	Posttest
	11:30–14:30	Lunch break
	14:30-	Going home

**Figure 4:**
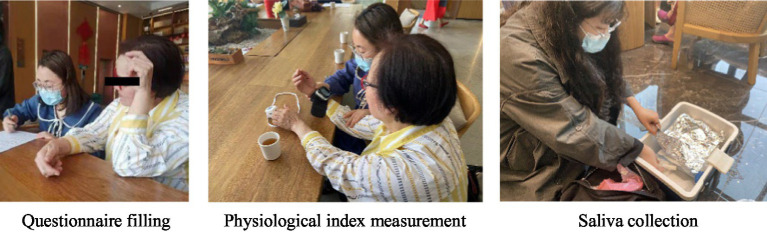
Indicator collection.

The pretest was conducted before lunch on Day 1 (10.30 am–11.30 am). After the participants rested quietly for 5 min, blood pressure, pulse, and finger oxygen saturation were measured, and saliva samples were collected using a saliva collection tube. After the measurement of physiological indicators, the sleep dysfunction rating scale (SDRS) was issued to evaluate the subjective sleep quality in the first 3 days of the experiment (Figure 4). The experimental schedule began after the pretest, and the staff will arrange daily meals, lodging and activities throughout the entire process. Posttests were scheduled at the same time on the third day to eliminate the effect of diurnal variations in circadian rhythms.

### 2.3. Physiological indicators

Changes in blood pressure (SBP, DBP) and pulse (P) were measured using an Omron wrist-type smart electronic sphygmomanometer (HEM-6322 T, Omron Dalian Co., Ltd.). Changes in finger blood oxygen (SpO2) saturation were measured by a Yuyue finger clip oximeter (YX306, Jiangsu Yuyue Medical Equipment Co., Ltd.). Saliva samples were collected using Salivette^®^ saliva collection tubes (Sarstedt, Germany). Salivary alpha-amylase (sAA) and salivary cortisol (SC) were detected using the Human sAA ELISA Kit (Cat. No.: ZC-54188) and Human SC ELISA Kit (Cat. No.: ZC-54504) kits (Shanghai Zhuocai Biotechnology Co., Ltd. company). The concentration of saliva indicators was determined using enzyme-linked immunosorbent assay (ELISA), and the microplate reader model SpectraMAX Plus384 (Meigu Molecular Instruments Co., Ltd.).

Before saliva sample collection (final stage of measurement), participants were well rested and acute stressful events were excluded as much as possible. Participants were required to abstain from food and drink for 30 min. A cotton swab was placed in the mouth for 2 min without chewing, and the cotton swab was returned to the collection tube after collection. The saliva samples were immediately frozen at −20°C and transported in ice boxes (27). Saliva was extracted by the centrifugal method for unified detection. Due to the diurnal variation in sAA and SC, the experimental schedule ended after lunch on the third day. We chose to collect data at the same time before lunch to rule out a possible morning sudden change in sAA and SC. Previous research revealed that sAA in older women dropped sharply 30 min after waking, rose steadily during the day, and peaked in the late afternoon. SC showed a circadian rhythm in which it rose sharply 30 min after waking up, decreased steadily during the day, and reached its lowest level at night. Simultaneously, changes in sAA and SC circadian rhythms mark the level of human sympathetic activity and psychosocial factors (risk factors, psychosocial resources, general health and well-being), respectively (28, 29).

### 2.4. Psychological indicators

Considering the reading comprehension ability of older people and experimental period, the shorter sleep dysfunction rating scale (SDRS) was used to assess the changes in sleep quality of the older people over the 3 days period. The SDRS is a quantitative assessment tool for insomnia severity based on the Chinese Classification and Diagnostic Criteria for Mental Disorders (3rd Edition) and has good reliability and validity in evaluating sleep quality, sleep duration, different clinical manifestations of insomnia, and insomnia-related discomfort (30). The scale has a total of 10 items, each item has a corresponding assessment guide, and the participants scored their symptoms on a scale from 0 to 4 points.

### 2.5. Statistical analysis

Statistical analysis of the data was performed using SPSS 26.0 (IBM Corp., Armonk, NY, United States). Single data or the difference before and after data were in line with normal distribution, paired *t*-test was used to compare the difference between pre- and posttest, and between-group (Winter/Spring, Normal/HTN, W-Normal/W-HTN, S-Normal/S-HTN) differences in pre- and posttest change values. Multivariate repeated measures analysis of variance was used to test the main effect (time (pre-/post-), season (winter/spring), health condition (normal/HTN)) and their interaction. The minimum significance test (LSD) was used to evaluate the significance. *p* < 0.05 was considered statistically significant.

## 3. Results

Repeated measures of variance for all indicators are shown in Table 3. Linpan nature therapy had a significant main effect on blood pressure, pulse, SpO2 and SDRS scores. The interaction effects between with or without hypertension and the therapy intervention was only significant for SBP, SC, and SDRS scores.

**Table 3:** Main and interaction effects of multivariate repeated treatments on the indicators.

Source	Indicator	SS	df	MS	*F*	*p*
A (pre-/post-)	SBP	2318.817	1	2318.817	50.934	0.000**
	DBP	1174.837	1	1174.837	31.167	0.000**
	P	294.817	1	294.817	12.873	0.001**
	SpO2	4.817	1	4.817	4.059	0.049*
	sAA	11525.893	1	11525.893	2.390	0.128
	SC	7.297	1	7.297	0.252	0.618
	SDRS	920.417	1	920.417	153.198	0.000**
A × B (Season)	SBP	48.600	1	48.600	1.068	0.306
	DBP	13.538	1	13.538	0.359	0.551
	P	84.017	1	84.017	3.669	0.061
	SpO2	2.400	1	2.400	2.023	0.161
	sAA	14437.360	1	14437.360	2.994	0.089
	SC	61.902	1	61.902	2.138	0.149
	SDRS	0.267	1	0.267	0.044	0.834
A × C (Health condition)	SBP	190.817	1	190.817	4.191	0.045*
	DBP	53.204	1	53.204	1.411	0.240
	P	0.150	1	0.150	0.007	0.936
	SpO2	0.817	1	0.817	0.688	0.410
	sAA	12300.164	1	12300.164	2.551	0.116
	SC	148.915	1	148.915	5.144	0.027*
	SDRS	33.750	1	33.750	5.617	0.021*
A × B × C	SBP	24.067	1	24.067	0.529	0.470
	DBP	1.838	1	1.838	0.049	0.826
	P	0.017	1	0.017	0.001	0.979
	SpO2	0.000	1	0.000	0.000	1.000
	sAA	10438.909	1	10438.909	2.165	0.147
	SC	66.210	1	66.210	2.287	0.136
	SDRS	9.600	1	9.600	1.598	0.211
Error	SBP	2549.450	56	45.526		
	DBP	2110.925	56	37.695		
	P	1282.500	56	22.902		
	SpO2	66.450	56	1.187		
	sAA	270015.008	56	4821.697		
	SC	1621.222	56	28.950		
	SDRS	336.450	56	6.008		

*p < 0.05 and **p < 0.01.

### 3.1. Blood pressure

**Figure 5:**
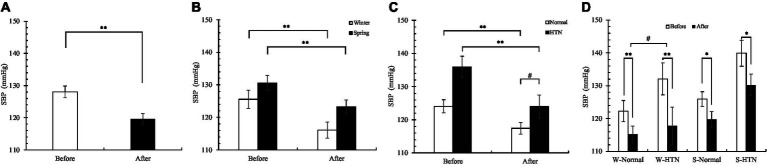
**(A)** Pre- and posttest changes in overall SBP; **(B)** Pre- and posttest changes in SBP in the winter and spring groups; **(C)** Pre- and posttest changes in SBP in the normal and HTN groups; **(D)** Pre- and posttest changes in SBP in the two-factor groups, *p < 0.05 and **p < 0.01, # Indicates that there is a significant difference between-group in the changes between the pre- and posttest, *p* < 0.05.

Overall, SBP decreased significantly in 60 participants (Figure 5A). The decline in the winter group (7.5%) was 1.8% higher than that in the spring group (5.7%) (Figure 5B). The reduction in the HTN group (8.8%) (before: 136 ± 3.2 mmHg; after: 124 ± 3.5 mmHg) was significantly higher than that in the normal group (5.4%) (before: 124.1 ± 1.97 mmHg; after: 117.45 ± 1.76 mmHg) (Figure 5C). Among the four groups divided by two factors, the W-HTN group (10.8%) (before: 132.1 ± 4.89 mmHg; after: 117.8 ± 5.69 mmHg) had the highest decrease, which was significantly higher than the W-Normal group (5.8%) (Figure 5D). This result indicated that the changes in systolic blood pressure of hypertensive patients who participated in the winter were most affected by nature therapy.

**Figure 6:**
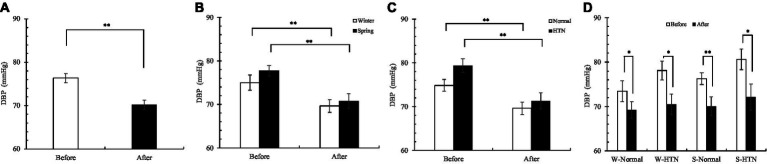
**(A)** Pre- and posttest changes in overall DBP; **(B)** Pre- and posttest changes in DBP in the winter and spring groups; **(C)** Pre- and posttest changes in DBP in the normal and HTN groups; **(D)** Pre- and posttest changes in DBP in the two-factor groups, *p < 0.05 and **p < 0.01.

Similar changes were observed in diastolic blood pressure. The reduction of the HTN group (before: 79.35 ± 1.56 mmHg; after: 71.3 ± 1.83 mmHg) (10.1%) was higher than that of the normal group (before: 74.85 ± 1.36 mmHg; after: 69.63 ± 1.41 mmHg) (7%) (Figure 6C). Unlike SBP, the reduction in the spring group (9%) was higher than that in the winter group (7.2%) (Figure 6B). Among the four groups divided by two factors, the S-HTN group (before: 80.6 ± 2.31 mmHg; after: 72.1 ± 2.94 mmHg) had the highest decrease (10.5%), which was higher than that of the W-Normal group (5.8%), the W-HTN group (9.7%) and the S-Normal group (8.1%) (Figure 6D). This result indicated that the DBP of hypertensive patients who participated in the spring was most affected by the cross-effect.

### 3.2. Pulse

**Figure 7:**
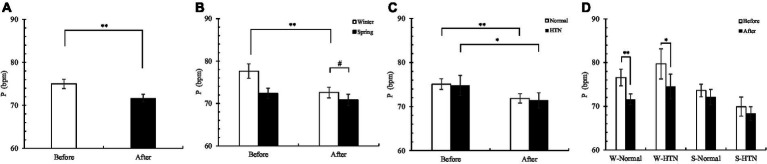
**(A)** Pre- and posttest changes in overall pulse; **(B)** Pre- and posttest changes in pulse in the winter and spring groups; **(C)** Pre- and posttest changes in pulse in the normal and HTN groups; **(D)** Pre- and posttest changes in pulse in the two-factor groups, *p < 0.05 and **p < 0.01.

Sixty participants showed a significant decrease in the overall pulse (Figure 7A). The decrease in the winter group (6.5%) was significantly higher than that in the spring group (2.1%) by 4.4% (Figure 7B), but no significant interaction effect was observed in Table 3 (probably related to sample size). The difference was that the reduction in the HTN group (before: 74.8 ± 2.29 bpm; after: 71.4 ± 1.74 bpm) (4.5%) was almost the same as that in the normal group (before: 75.1 ± 1.21 bpm; after: 71.85 ± 1.06 bpm) (4.3%) (Figure 7C). Among the four groups divided by two factors, the decrease in the W-Normal group (Before: 76.6 ± 1.92 bpm; After: 71.6 ± 1.23 bpm) (6.5%) and the W-HTN group (Before: 79.7 ± 3.47 bpm; After: 74.5 ± 74.5 ± 2.84 bpm) (6.5%) was almost the same. The reduction in the S-Normal group (before: 73.6 ± 1.45 bpm; after: 72.1 ± 1.77 bpm) (2%) and S-HTN group (before: 69.9 ± 2.18 bpm; after: 68.3 ± 1.59 bpm) (2.3%) was also almost the same (Figure 7D). We believe that hypertension is more related to SBP (31) and less related to pulse. Pulse changes are more affected by seasonal factors.

### 3.3. Finger oxygen saturation

**Figure 8:**
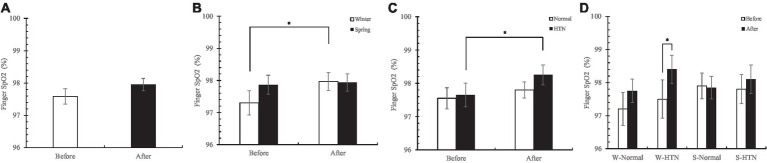
**(A)** Pre- and posttest changes in overall finger SpO2; **(B)** Pre- and posttest changes in finger SpO2 in the winter and spring groups; **(C)** Pre- and posttest changes in finger SpO2 in the normal and HTN groups; **(D)** Pre- and posttest changes in finger SpO2 in the two-factor groups, *p < 0.05.

Overall, finger SpO2 increased in 60 participants (Figure 8A). The increase in the winter group (0.7%) was higher than that in the spring group (0.1%) (Figure 8B). The increase in the HTN group (0.6%) was higher than that in the normal group (0.3%) (Figure 8C). Among the four groups divided by two factors, the W-HTN group (before: 97.5 ± 0.58%; after: 98.4 ± 0.43%) had the highest increase (0.9%). This suggests that the nature therapy on finger SpO2 was the greatest in patients with HTN who participated in the winter (Figure 8D).

### 3.4. Salivary alpha-amylase

**Figure 9:**
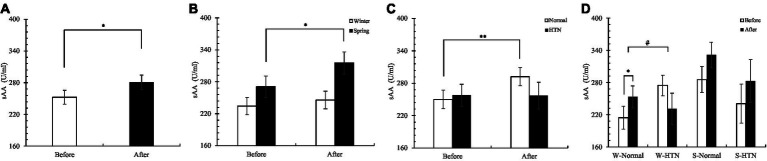
**(A)** Pre- and posttest changes in overall sAA; **(B)** Pre- and posttest changes in sAA in the winter and spring groups; **(C)** Pre- and posttest changes in sAA in the normal and HTN groups; **(D)** Pre- and posttest changes in sAA in the two-factor groups, *p < 0.05 and **p < 0.01, # Indicates that there is a significant difference between-group in the changes between the pre- and posttest, *p* < 0.05.

Overall, sAA concentrations were significantly elevated in 60 participants (Figure 9A). The increase in the winter group (4.8%) was 11.7% lower than that in the spring group (16.5%) (Figure 9B). Notably, the normal group significantly increased (16.9%) (before: 249.98 ± 17.04 U/mL; after: 292.24 ± 16.78 U/mL), but the HTN group decreased slightly (0.3%) (before: 257.6 ± 20.32 U/mL; after: 256.91 ± 24.8 U/mL) (Figure 9C). Among the four groups divided by two factors, we observed that the group leading to the above differences appeared in the winter HTN group (before: 274.61 ± 19.12 U/mL; After: 230.87 ± 29.21 U/mL). Its sAA concentration decreased by 15.9%, and the other three groups all increased. Meanwhile, there were significant differences between the W-Normal group (before: 214.36 ± 21.42 U/mL; After: 253.15 ± 20.6 U/mL) and W-HTN group. This shows that in winter, HTN has a significant effect on the sAA concentration. We hypothesized that sAA secretion in hypertensive patients participating in the winter was inhibited by the nature therapy (Figure 9D).

### 3.5. Salivary cortisol

**Figure 10:**
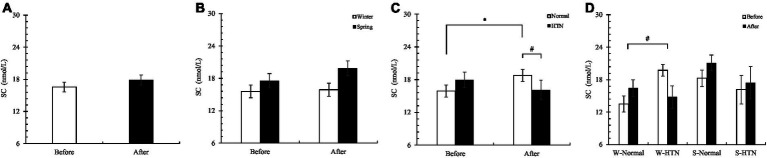
**(A)** Pre- and posttest changes in overall SC; **(B)** Pre- and posttest changes in SC in the winter and spring groups; **(C)** Pre- and posttest changes in SC in the normal and HTN groups; **(D)** Pre- and posttest changes in SC in the two-factor groups, *p < 0.05, # Indicates that there is a significant difference between-group in the changes between the pre- and posttest, *p* < 0.05.

Changes in SC were consistent with sAA, with an increase in overall SC concentration in 60 participants (Figure 10A). The increase in the winter group (2%) was 11.2% lower than that in the spring group (13.2%) (Figure 10B). The normal group (before: 15.89 ± 1.13 nmol/L; after: 18.77 ± 1.1 nmol/L) increased significantly, and the HTN group decreased (before: 17.94 ± 1.44 nmol/L; after: 16.1 ± 1.79 nmol/L). There was a significant difference between the two groups, indicating that the presence or absence of hypertension had a significant effect on the changes in SC concentration (Figure 10C). Among the four groups divided by two factors, the W-HTN group (before: 19.72 ± 1.08 nmol/L; after: 14.78 ± 2.06 nmol/L) decreased by 25.1%, while the other three groups increased. There was a significant difference between the W-normal group (before: 13.51 ± 1.5 nmol/L; after: 16.45 ± 1.51 nmol/L) and the W-HTN group (Figure 10D). This shows that in winter, the presence or absence of hypertension has a significant effect on SC concentration. Like sAA, SC secretion may also be inhibited depending on the cross-effect.

### 3.6. Sleep dysfunction rating score

**Figure 11:**
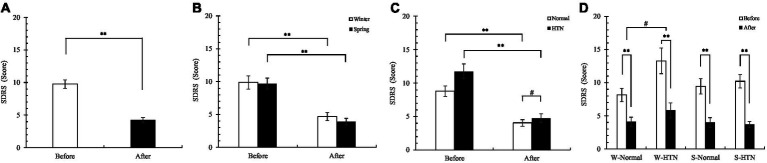
**(A)** Pre- and posttest changes in overall SDRS score; **(B)** Pre- and posttest changes in SDRS score in the winter and spring groups; **(C)** Pre- and posttest changes in SDRS score in the normal and HTN groups; **(D)** Pre- and posttest changes in SDRS score in the two-factor groups, *p < 0.05 and **p < 0.01, # Indicates that there is a significant difference between-group in the changes between the pre- and post-test, *p* < 0.05.

Overall, SDRS scores decreased significantly in 60 participants (Figure 11A). The decrease in the winter group (52.7%) was lower than that in the spring group by 7.1% (59.8%) (Figure 11B). The reduction of the HTN group (before: 11.75 ± 1.12 Score; After: 4.75 ± 0.64 score) (59.6%) was significantly higher than that of the normal group (before: 8.8 ± 0.76; after: 4.05 ± 0.49) (54%), indicating that the improvement in sleep disturbance in the HTN group was significantly higher than that in the normal group (Figure 11C). Among the four groups divided by two factors, the S-HTN group (before: 10.2 ± 1.01; after: 3.7 ± 0.47 Score) had the highest decrease (63.7%), which was higher than the W-Normal group (49.7%) and the W-HTN group (56.4%) and S-Normal group (57.7%). This result indicated that the improvement in sleep disturbance in hypertensive patients who participated in the spring nature therapy was most pronounced. The reduction in the W-HTN group (before: 13.3 ± 1.93 Score; After: 5.8 ± 1.13 score) was significantly higher than that in the W-Normal group (before: 8.15 ± 0.99 Score; After: 4.1 ± 0.7 Score). This indicated that in winter, the improvement in sleep disturbance in hypertensive patients was significantly higher than that in healthy subjects but less than in spring participants (Figure 11D).

## 4. Discussion

### 4.1. Effects of Linpan nature therapy on the cardiovascular system and sleep quality in older women

In this study, Linpan nature therapy significantly reduced blood pressure in older women. For the normal group with healthy blood pressure, Linpan nature therapy regulates blood pressure to prevent cardiovascular disease. Studies have shown that forest therapy can reduce elevated blood pressure in the middle-aged and older people to an optimal range and may prevent healthy blood pressure from developing into HTN (12). For older hypertensive patients, forest therapy for days or even weeks inhibits the renin-angiotensin system and inflammation, thereby preventing cardio-vascular disease. Forest therapy can reduce blood pressure in older hypertensive patients and assist in the treatment of HTN (16, 32). Our results are consistent with this finding; the blood pressure drop in the HTN group was higher than that in the normal group, indicating that Linpan nature therapy has a more significant antihypertensive effect on older hypertensive women. Notably, reducing SBP to less than 120 mmHg significantly reduces the probability of cardiovascular events and mortality (33). Song et al.’s study also specifically focused on participants with a systolic blood pressure above 120 mmHg (34). In our study, the mean SBP in the normal group was adjusted from 124.1 ± 1.97 mmHg to 117.45 ± 1.76 mmHg, indicating that not only the blood pressure of older hypertensive women was significantly relieved, but the normal group of women with high systolic blood pressure also experienced health benefits. In conclusion, Linpan nature therapy does have a significant improvement effect on the cardiovascular system of older women, especially older women with HTN. Considering that forest therapy is an effective adjuvant therapy for cardiovascular disease (17), we believe that Linpan nature therapy can also be an adjunct in the treatment of cardiovascular disease. In this experiment, more blood pressure drops and blood oxygen increases were observed in the winter than in the spring (*p* > 0.05). Research indicates that cold increases sympathetic tone, which increases heart rate and blood pressure, thereby increasing myocardial oxygen demand (35). Moreover, the blood pressure of the older people is higher in the winter than in the summer, and the change is larger (36). This may be the reason winter participants received more health benefits than the spring group after Linpan nature therapy.

Linpan nature therapy can significantly reduce SDRS scores and improve sleep quality in older women. We reviewed several previous studies that reported that forest therapy increased participants’ salivary melatonin levels (37), with positive therapeutic effects on mood and sleep (38), especially in postmenopausal women (39). This result is consistent with ours and suggests that future studies should investigate whether natural remedies increase salivary melatonin secretion, thereby improving sleep quality. Studies have shown that sleep disturbances are associated with increased blood pressure and the risk of HTN (40), and improving insomnia can significantly help lower blood pressure in hypertensive insomnia patients (41). We also found that older women with HTN had better sleep quality improvements and lower blood pressure than the normal group. However, the specific mechanism of this result needs further study. Among the different results between the winter and spring groups, the warmer spring group experienced a greater improvement in sleep quality. Studies have shown that in the summer, compared with the autumn and winter, the older people have less sleep time and increased arousals. In autumn and winter, the temperature and humidity of the sleeping environment are low, and arousal is reduced, thereby prolonging sleep time (42). We speculate that in the Linpan environment of this study, the spring sleep microclimate maybe more in line with the physical comfort of older people. This suggests that future research needs to further explore these differences during different seasons, and collect data on the sleep environment microclimate. Improvement in sleep disturbance in older women with HTN in the winter was significantly higher than that in healthy people. We speculate that hypertensive factors and winter factors interact to influence the physiological mechanism. The interaction effect reduced arousals more, resulting in better sleep in the winter hypertensive group than in the winter normal group.

### 4.2. Effects of Linpan nature therapy on endocrine function and psychological stress in older women

In this study, the sAA and SC concentrations rose after the experiment. This is in contrast to previous findings, which we speculate may be related to the activities performed by the participants. Previous research has focused on exposure to the natural environment and engaging in light, nonexercise activities to relieve stress. Such as walking in the forest and sitting and watching the view (11, 43). The study found that sAA decreased after forest walking relaxation and gardening activities in middle-aged and older people, but there was no significant effect (22, 44). Ochiai et al. (10) also studied SC levels and found that cortisol levels were reduced when subjects participated in forest therapy. The activities involved in the above study were mainly walking, sitting or lying down, with low intensity.

sAA is considered to be a sensitive biomarker reflecting sympathetic nervous system (SNS) activity and may be on an uptick during psychological stress (45). The response speed of sAA is faster than that of hormones such as cortisol (46). We believe that the Linpan environment and gregarious social model differ from the urban living environment in that it increases the excitement of the participants and provides more social interaction. Among them, negative and positive interactions can affect stress and mood, and positive interactions can increase people’s arousals and expectations, thereby affecting the changes in sAA (47). The study found that patients with mild AD had a significant increase in well-being scores after music therapy, and sAA also increased after treatment. They suggested that increased sAA could serve as a possible marker of increased well-being in AD patients (26). Therefore, we speculate that the increase in sAA after nature therapy may have been caused by the same reasons as above.

Previous research found that gardening activity-oriented forest therapy improved cognitive performance in older people with MCI (38). For the participants’ schedules, we chose moderate-intensity sports such as dancing, gateball, field exercise, and Baduanjin/Tai Chi. The participants had a variety of activity patterns and a higher intensity of exercise than in common forest therapy, and exercise can upregulate sAA (48). Studies have shown that moderate-intensity exercise can increase sAA and thus affect cognitive performance (49, 50). A certain intensity of aerobic exercise can also activate the memory ability of older people with memory impairment, and the sAA concentration increased after the experiment. This suggests that exercise can intervene to treat cognitive decline in older people (51). The LC (locus coeruleus)-NE (norepinephrine) system plays an important role in arousal, attention, and stress responses (52), while sAA is an indicator of central NE activation (53), showing an upward trend when NE is activated (51). We speculate that the increase in sAA concentration in this project maybe due to exercise-activated LC resulting in increased brain NE release (51). Perhaps Linpan nature therapy has the potential to activate cognitive and memory abilities, and increase the body’s arousal response of older women. Unfortunately, cognitive function was not assessed in this program, but there is a reference value from the previous study for interpreting the results of sAA and SC in this experiment.

SC is often used as a biomarker of psychological stress and is secreted by the hypothalamic–pituitary–adrenal axis (HPA) (54). SC is involved in various physiological regulatory processes in the body and is related to cognitive ability (55). In this study, the increase in SC concentration may be related to the negative feedback regulation mechanism of the participants and the dysregulation of the HPA axis. We also found low levels of pretest values in a minority of participants, and studies suggest that too little SC secretion can contribute to Addison’s disease (56). The normal circadian rhythm of SC in older people can be maintained, while daily variability may be increased (57). This suggests that Linpan nature therapy may correct participants with mild HPA axis dysregulation, but future research regarding these individuals is necessary.

Notably, there was consistency in the changes in sAA and SC. They showed rising results overall, with significant differences in the winter group with or without HTN (Figures 9D, 10D). We speculate that this is related to seasonal differences between the winter and spring. Research has shown that employees have more mental health problems and lower mucosal immunity in the winter than in the summer (58). The researchers believe that for some perceived effects, the response of the HPA axis is more adaptive in winter, so the concentration of sAA-AR and C-AR (a calculation method of sAA and SC) in employees is lower in winter (58). Therefore, we speculate that the decrease in sAA and SC concentrations in the W-HTN group may be related to the sensitivity of the human HPA axis in the winter. Older women with HTN had more suppression of HPA axis sensitivity in winter, causing them to be less responsive to stress. This may be due to the decreased immunity caused by HTN and winter and may also be related to the easier contraction of respiratory tract infections in the winter (58).

### 4.3. Limitations

The study is tentatively unclear about the influence of Linpan location on the effects of therapy, and there may be differences in the environment, experience, etc. caused by different Linpan locations. It was also not clear whether the level of social engagement, single activity, or intensity of activities were responsible for the changes in indicators during therapy.

The circadian and acute stress mechanisms in sAA and SC means that the time of day must be taken into account. A single salivary measurement does not have adequate between visit reliability, and in prior studies were collected usually at 8 a.m. or late at night (59). Given the realities of the participants, we chose to measure at the time after peak, which may not be biologically rigorous enough. Therefore, repeated collections on each sampling day better capture the circadian, allowing the establishment of diurnal trajectories of the study population to better interpret results (60).

The extrapolation for cognitive function was not based on relevant data, because sAA and SC did not decrease as a stress value, but showed another interesting result. The generation of this result may be related to the presence of individuals with cognitive decline among older participants (e.g., AD, MCI patients), and prior assessment of participants is needed in the future. The discussion of sleep improvement and seasonal temperatures is not based on relevant data, and factors besides temperature may also cause seasonal differences in sleep quality. In addition, no significant seasonal differences and Season-Therapy interaction effects were observed, which may require increasing the sample size.

## 5. Conclusion

Linpan, located in the suburbs, as an important part of urban green space, provides a valuable opportunity to improve the physical and mental health of urban residents. Our research suggests that Linpan nature therapy based on a variety of health activities can regulate the blood pressure, pulse and finger oxygen saturation of older women and relieve cardiovascular diseases. It significantly reduced SDRS scores and improved overall sleep quality. Especially in older women with hypertension. It may activate cognitive and memory functions to a certain extent and improve the body’s arousal response, but further research is required to prove this.

## Data availability statement

The raw data supporting the conclusions of this article will be made available by the authors, without undue reservation.

## Ethics statement

The studies involving humans were approved by the local Ethics Committee of the College of Landscape Architecture, Sichuan Agricultural University, China (protocol code SICAU201504120023 and date of 8 April 2015 approval). The studies were conducted in accordance with the local legislation and institutional requirements. The participants provided their written informed consent to participate in this study. Written informed consent was obtained from the individual(s) for the publication of any potentially identifiable images or data included in this article.

## Author contributions

XY, ZD, and MJ: conceptualization. XY and ML: investigation. XY, ZD, MJ, ZL, and ML: experimental design and progress. XY and MJ: data curation and analysis. XY: writing-original draft preparation. MJ, HT, and YW: writing-review and editing. ZD and ZL: funding acquisition. ZD and MJ: project administration. ZD, XM, and YF: resources. XH: validation and review. LD: supervision. All authors contributed to the article and approved the submitted version.
